# Why Are Cytomegalovirus-Encoded G-Protein-Coupled Receptors Essential for Infection but Only Variably Conserved?

**DOI:** 10.3390/pathogens14030245

**Published:** 2025-03-03

**Authors:** Suzan Fares, Benjamin A. Krishna

**Affiliations:** 1 Occlutech Holding AG, Feldstrasse 22, 8200 Schaffhausen, Switzerland; suzan.fares@occlutech.com; 2 Cambridge Institute of Therapeutic Immunology & Infectious Disease (CITIID), Cambridge CB2 0AW, UK; 3 Department of Medicine, University of Cambridge, Cambridge CB2 0QQ, UK

**Keywords:** cytomegalovirus (CMV), G-protein coupled receptors (GPCRs), signalling, animal models, therapeutics

## Abstract

Cytomegaloviruses (CMVs) encode viral G-protein-coupled receptors (vGPCRs) that have diverged from their cellular homologues to perform new functions. Human cytomegalovirus (HCMV) encodes four vGPCRs: UL33, UL78, US27, and US28, which contribute to viral pathogenesis, cellular signalling, and latency. While the role of US28 in chemokine signalling and viral latency is well characterised, the functions of other vGPCRs remain incompletely understood. Rodent cytomegaloviruses only have homologues to UL33 and UL78, while primates have two to five additional GPCRs which are homologues of US27 and US28. Different CMVs appear to have evolved vGPCRs with functions specific to infection of their respective host. As non-human CMVs are used as model organisms to understand clinical cytomegalovirus disease and develop vaccines and antivirals, understanding the differences between these vGPCRs helps researchers understand critical differences between their models. This review aims to address the differences between CMV vGPCRs, and how these differences may affect models of CMV disease to facilitate future research.

## 1. Introduction

Cytomegaloviruses (CMVs) are betaherpesviruses which infect a diverse range of organisms in a species-specific manner [1,2]. This species specificity has arisen due to the divergent evolution of cytomegaloviruses with their respective hosts across millions of years [3]. During this time, cytomegaloviruses have acquired copies of genes from their host, thereby encoding homologues. These homologues include cytokines [4], ribonucleotide reductase [5], major histocompatibility (MHC) proteins [6], and G-protein-coupled receptors (GPCRs), the subject of this review.

GPCRs are a large superfamily of seven transmembrane domain proteins, which play roles in vision (such as the archetypical rhodopsin), taste, smell, and neurotransmission [7]. CMVs encode homologues of chemokine receptors which are type-A, rhodopsin-like GPCRs, and are characterised by a hallmark outward movement of transmembrane 6 during protein activation [8]. These receptors are found throughout the immune system and bind chemokines, which signal to induce chemotaxis and cellular differentiation [9]. Although not the main focus on this review, GPCRs are targets for 30–40% of current drugs [10], and the study of viral GPCRs could therefore lead to novel antiviral drugs.

**Figure 1:**
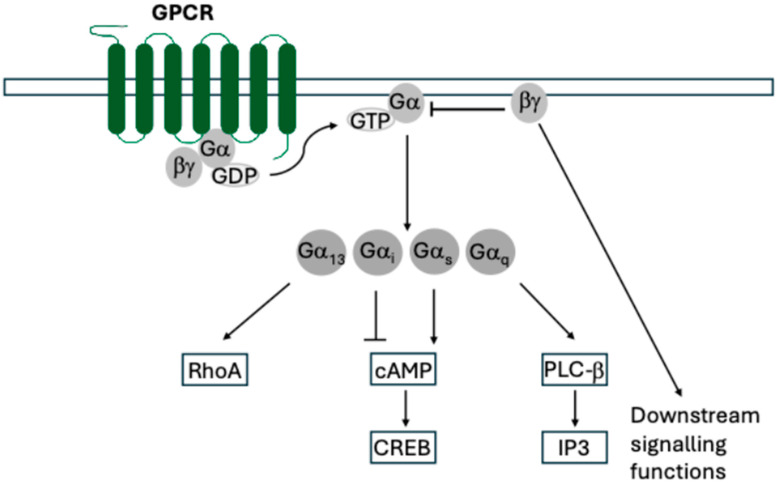
General diagram of GPCR functions. Upon binding ligands, GPCRs exchange GDP for GTP on the associated Gα protein, leading to dissociation and lateral diffusion of Gα and Gβ/γ. Both complexes have downstream signalling functions with Gβ/γ also acting to sequester and inhibit Gα.

GPCRs are coupled to a Gα/β/γ protein complex, and mediate signal transduction through the exchange of a GDP molecule with GTP on the coupled Gα protein (Figure 1). The Gα protein and Gβ/γ dimer then dissociate and diffuse laterally across the plasma membrane. Gα proteins canonically activate secondary messengers while Gβ/γ canonically inhibits Gα but can also signal too. There are multiple subfamilies of Gα proteins, categorised by sequence similarity, which include Gαi, Gαq, and Gαs, but host chemokine receptors signal via Gαi [11].

Despite being chemokine receptor homologues, the CMV-encoded GPCRs have diversified in function away from the host. CMV GPCRs tend to have signalling functions, but the importance of their signalling roles could still be better defined. Only one CMV GPCR, unique short (US)28, is known to bind extracellular chemokines, with others instead signalling constitutively, or potentially not at all. The roles of these CMV GPCRs have also diversified; some can still induce cellular chemotaxis but others play roles in viral replication, immune evasion, and latency/reactivation [12]. Additionally, some CMV GPCRs can bind multiple chemokine families, which is not seen by host chemokine receptors, and they can then signal via a range of Gα proteins [11]. Overall, this diversification of CMV GPCRs has created a class of proteins which modulate the host cell environment to favour viral replication and survival.

## 2. Conservation and Diversity of Cytomegalovirus GPCRs

Perhaps the most interesting aspect of the CMV GPCRs is that CMVs encode different numbers of GPCRs, and therefore, not all homologues are found in all CMVs and some CMVs encode multiple copies of some GPCRs. This raises the question of what functions these different CMV GPCRs serve in different hosts and will likely provide valuable insights into different host–virus interactions across the family of CMVs.

Human cytomegalovirus (HCMV) encodes four GPCRs: unique long (UL)33, UL78, US27, and US28 [13], which will act as the basis for this review. UL33 and UL78 are conserved among multiple mammalian CMVs, while US27 and US28 exist only in primate CMVs. This suggests a gene duplication event occurred in a common CMV ancestor, around the emergence of the primate order. This gene duplication could have occurred either from an ancestor of UL33/UL78 or from a host GPCR [12,13,14].

The HCMV-encoded GPCRs show remarkable differences between each other. In contrast to US28, which binds several human chemokines, the ligands for UL33, UL78, and US27 have not been identified [15,16]. Only UL33 and US28 have been shown to couple to Gα proteins and mediate Gα protein-dependent signalling [16,17,18], and while UL33 appears to only signal constitutively (ligand-independent), US28 can signal in both constitutive and ligand-dependent manners. US27 signals via Gβ/γ instead of Gα.

Many aspects of HCMV infections are difficult to replicate in vitro, including organ-level infection, immune interactions, and transmission in utero. Researchers therefore use animal models, infecting model organisms with their respective cytomegaloviruses, as HCMV is unable to replicate in these animals [19]. Mouse CMV (MCMV) has been used as a model for systemic CMV infection and uncovered several viral mechanisms involved in immunomodulation and viral dissemination [19,20,21,22]. Acute CMV in immunocompetent mice is characterised by robust viral replication in internal organs (such as the liver and spleen) within 2 to 3 days postinfection, followed by secondary virus dissemination to the salivary gland. It is presumed that the virus produced in the spleen is the virus that ultimately reaches the secondary sites such as salivary glands [19,23,24]. The virus can be detected in the saliva of both humans and mice for prolonged periods of time, and therefore, the salivary glands are likely important sites for horizontal transmission [19,25,26,27]. MCMV can persist in the salivary glands for months before eventually being cleared by a cluster of differentiation (CD)4+T cell responses [19,28,29]. Guinea pig CMV (GpCMV) and rhesus macaque CMV (RhCMV) are both used largely to study maternal–foetal CMV transmission, towards developing effective HCMV vaccines [30].

Understanding the differences between CMVs and their interactions with their hosts can inform which aspects of animal models are accurate and where limitations lie. As the viral GPCRs show major variability between CMV species, understanding these differences can guide our understanding of host–CMV interactions and the models we use.

## 3. Human Cytomegalovirus UL33 and Its Homologues

UL33 shows the closest homology to CC-chemokine receptor (CCR)3 and CCR10 [31] and currently appears to play two separate roles in CMV biology, which have not yet been linked. Firstly, UL33 and its homologues facilitate viral spread in specific cell types, which has been observed in in-vitro and in-rodent models. Additionally, UL33 has ligand-independent signalling functions, which vary somewhat between HCMV-encoded UL33, R33 and M33.

### 3.1. UL33 and Its Homologues Facilitate the Infection of Specific Cell Types and Organs, Which Vary Between Model Organisms

UL33 is expressed with true late kinetics [32] and plays a role in cell–cell spread in fibroblasts, but only in the Merlin strain of HCMV [33] and not in TB40E [34] or AD169 [35]. This difference may be due to Merlin preferentially spreading via the cell–cell route and. As Merlin likely better reflects clinical strains of HCMV [36], this may therefore reflect a physiologically relevant role for UL33 for viral spread in vivo. The mechanism for facilitating cell spread remains unclear, but UL33 gene deletion did not affect the expression of other HCMV proteins such as immediate early 1 (IE1), phosphoprotein (pp)28, glycoprotein B, or US28 [33], suggesting UL33 is not required to drive viral lytic infection. Merlin-encoded UL33 has a range of amino acid point mutations compared to AD169 and TB40/E, which may also change protein functions between viral strains. Identifying these differences may help explain why these strains show different phenotypes. In TB40/E, UL33 is required for replication in epithelial cells [37].

The HCMV UL33 gene is conserved among all betaherpesviruses. The murine cytomegalovirus (MCMV) and rat cytomegalovirus (RCMV) UL33 orthologs (M33 and R33, respectively) are both essential for viral replication within the host’s salivary glands and contribute to pathogenesis in vivo [19,38,39,40,41,42]. M33 promotes MCMV infection in heart tissue [43]. However, both M33 and R33 proteins are dispensable for virus replication in fibroblasts [38,39,40,41,42]. The GpCMV homologue GP33 was not required for viral pathogenesis in normal guinea pigs, but did contribute to viral load in guinea pig dams (pregnant female guinea pigs) in the spleen, liver, and placenta [44]. GP33 may also contribute to viral dissemination via the salivary glands as with MCMV and RCMV but as the bacterial artificial chromosome (BAC)-derived GpCMV used does not spread to the salivary glands, this has not yet been shown [44].

These results are similar to the role of UL33 in the viral spread of Merlin in fibroblasts [33] and TB40/E in epithelial cells [37]. The similarities in the phenotype here suggest that the UL33/M33/R33 family have maintained a function supporting cell-type-specific replication and viral pathogenesis throughout CMV speciation. Further support for this hypothesis comes from the sequencing of clinical HCMV samples, which showed extensive mutations in the extracellular loops but not the intracellular loops [45]. This is consistent with a vital signalling role without specific cytokine binding.

### 3.2. UL33 and Its Homologues Also Signal Constitutively

UL33 and its homologues also signal constitutively, in a ligand-independent manner. UL33 couples to Gαq/Gαi and Gαs to produce inositol phosphate (InsP) and activate cyclic AMP response element binding protein (CREB), respectively [17,18]. This constitutive activation of CREB plays a role in HCMV reactivation from latency by driving major immediate early promoter (MIEP) expression in THP-1 and Kasumi-3 models of latency [34]. As UL33 signals constitutively, how this activity is controlled to prevent MIEP activation during latency remains unclear. Possibilities include a currently unidentified agonist or antagonist ligand, increases in pUL33 expression, or interaction with another HCMV protein. pUS28 does interact with pUL33, showing reduced pUS28-mediated activation of nuclear factor kappa light-chain enhancer of activated B cells (NF-κB) [46]. Whether similar interactions occur during latency remains unclear. Apart from reactivation from latency, UL33 constitutive signalling enhances tumour growth via constitutive activation of the signal transducer and activator of the transcription (STAT)3 proangiogenic pathway to CREB, NF-κB, and serum response factor (SRF) [47].

M33 appears to have similar signalling capabilities to UL33: activating Gαq/Gα11 phospholipase C (PLC-β), protein kinase C (PKC), NF-κB, nuclear factor of activated T cells (NF-AT), and CREB transcription factors [19,48,49]. This activation happens in the absence of ligands, i.e., constitutively [18,19]. Interestingly, although M33 is more similar in sequence to UL33 compared to US28 [18], US28 is sometimes described as a functional homologue of M33, as US28 and M33 both activate NF-κB and CREB in Cos-7 cells, while UL33 only activates CREB [18]. GP33 behaves similarly to M33, activating CREB and NF-AT [44].

R33 appears to signal quite differently to its viral homologues. Although signalling constitutively [42], R33 couples to the Gαq class of G proteins and activates PLC [42]. In addition, R33 partially activates Gαi, leading to a constitutive inhibition of CREB [17,42]. In that manner, R33 differs from UL33, M33, and GP33, which enhance CREB [17]. RCMV R33 is also a potent activator of Gαq/11, while HCMV UL33 was a weak activator of Gαq/11 in some studies [17,18].

### 3.3. UL33 and Its Homologues—Questions Yet to Be Answered

Case et al. showed in 2008 that M33 is required for replication in NIH3T3 cells, mouse embryonic fibroblasts (MEFs), and the salivary gland of BALB/C mice, but that the signalling functions of M33 are not [49]. This raises the question of why these proteins have retained signalling function, and are not instead signalling-dead—as is thought for UL78. One possibility is that R33/M33/GP33 signal to facilitate reactivation from latency, like UL33. As M33 and GP33 both activate CREB, which activates the MIEP and drives HCMV reactivation, this appears plausible. However, R33 appears to serve the exact opposite function. It could be that R33 signals differently in different cell types, as has been shown for US28 [50], or it could be that RCMV reactivates using different signalling pathways from other rodent (and human) CMVs.

The RhCMV and GMCMV homologues of UL33 have no known functions.

## 4. Human Cytomegalovirus UL78 and Its Homologues

UL78 is expressed with early kinetics [51], and transcripts have been detected in some [52] but not all [53,54] latency models. UL78 has reduced sequence similarity to other GPCRs, leading to its later identification as an HCMV-encoded GPCR [12,55,56]. UL78 is classified as an orphan receptor [33,57], and consistent with this, cryo-electron microscopy (EM) analysis revealed that the chemokine binding pocket is occluded by an inward-folded extracellular loop 2 [56]. The same paper revealed a unique extracellular conformation for UL78, where extracellular architecture is more compact and lacks the participation of extracellular loop-3 (ECL3) or interaction with the N-terminus.

UL78 is dispensable for replication in fibroblasts or an ex vivo renal artery organ culture [51] but required for viral entry in endothelial and epithelial cells [58]. The precise mechanism by which UL78 contributes to viral entry is not yet known but UL78 protein is present in the infectious virion [58]. UL78 has no described signalling activity and consistent with this, intracellular loop (ICL) 2 and the extensive C terminal domain of the receptor cover the Gα_i_ binding site on the intracellular face of the GPCR [56]. In addition to this, UL78 does not have the classic DRY motif in its active site [59] but instead has DLR, which may reduce or ablate Gα protein activation.

The structure of UL78 was solved by cryo-EM as a trimer [56], and consistent with this, UL78 interacts in the membrane with other GPCRs. The pUS28:pUL78 dimer reduces pUS28-mediated activation of NF-κB [46]. UL78 forms heteromers with CCR5 and CXC-chemokine receptor (CXCR)4, which predominantly negatively affect a plethora of functions like cell surface expression, ligand-induced internalisation, and signal transduction [60,61].

The UL78 gene is highly conserved, both among clinical isolates of HCMV [51], and among other CMVs, including all rodent and primate CMVs [62], suggesting that it plays a less species-specific role than US27 or US28. Consistent with this, the deletion of M78 from MCMV resulted in the attenuation of replication in mouse fibroblast and macrophage cell lines in vitro [62], while the deletion of R78 from rCMV attenuated in vitro replication in rat embryo fibroblasts, and reduced viral pathogenesis and replication in the spleen [38,39]. Although not showing an identical phenotype to HCMV UL78, which is not required for replication in fibroblasts, the M78 and R78 homologues clearly play a role in cell spread and viral dissemination, making them critical for the viral life cycle. GP78 does not yet have a defined function [44,63], while Rh107, the RhCMV homologue, is non-essential and can be removed for the development of CMV-based vaccines [64,65].

How UL78 and its homologues achieve this at the molecular level is less well explained. M78 undergoes constitutive endocytosis [41,66], a phenomenon that is mainly dependent on the C terminus of the receptor [67]. M78 also contributes to MHC Class II degradation in RAW-C2TA mouse macrophage cells [68]. R78 prevents the formation of syncytia in fibroblasts, but why this occurs remains unclear. The presence of these viral GPCRs in the virion [58,62] and their role supporting immediate early gene expression or viral entry [58,62], but without signalling capacity, suggest perhaps that they contribute to the virion structure in a way that facilitates entry.

## 5. Human Cytomegalovirus US27 and Its Homologues

US27 is expressed during lytic infection with early–late [69] kinetics, and is the only HCMV-encoded GPCR with no evidence of expression during latent infection [52]. Indeed, US27 mRNA levels are expressed at much lower levels than US28 in Kasumi-3 and CD34^+^ in vitro latency models [70]. As US27 and US28 are expressed during lytic infection as a polycistronic transcript [71,72], it is unclear how the US28 transcript is expressed alone during latency, or why US28 protein expression shows phosphonoformic acid (PFA)-independent early kinetics, while US27 shows PFA-dependent true late kinetics of expression [32]. Either alternative splicing, separate monocistronic mRNAs, or translation-level control must be at play.

US27 encodes a heavily glycosylated [69] orphan receptor [60,73], which shows about 30% homology to chemokine receptors [12,56,74]. Cryo-EM structures show that US27 has an occluded ligand-binding pocket and so cannot bind ligands and captures a guanosine diphosphate-bound inactive Gα_i_, suggesting that the receptor is constitutively unable to signal [12,56,57].

US27, like UL33, contributes to the lytic infection of fibroblasts but is not essential for growth [75], facilitating viral extracellular spread, not cell–cell spread [76]. This appears to be the case for the clinical isolates FIX and TB40/E [76], but not the more laboratory-passaged isolates AD169 or Towne [77].

US27 modulates the expression and subsequent activity of CXCR4 [73,75,78,79]. CXCR4 is a human-encoded chemokine receptor which binds CXCL12 and signals via both Gα_i_-Akt-MEK-ERK (extracellular signal-regulated kinase) and PI3K-IP3/DAG. Consistent with structural analysis, suggesting US27 binds inactive Gα_i_, the protein signals via Gβ/γ. CXCR4 plays roles in cell growth [80], chemotaxis [81], and stem cell homeostasis [82]. CXCR4 mRNA is induced by US27 as early as 3 h post-infection, indicating that US27 carried by the virion mediates this effect, US27 upregulates transcription via constitutive activation of the transcription factor nuclear respiratory factor 1 (NRF-1), via Gβ/γ activation of phosphoinositide 3-kinase (PI3K) rather than Gα [75]. Additionally, US27 colocalises with and enhances endocytosis of CXCR4 upon CXCL12 binding, which slows the recycling of CXCR4 back to the cell surface [78]. Sequencing analysis of US27 from clinical HCMV samples shows occasional deletion of the N terminal region, suggesting that US27 may not require cytokine binding for HCMV replication [83].

These effects on CXCR4 were proposed by the authors to direct infected cells to areas with high CXCL12 secretion, including bone marrow stromal tissue, which would contribute to viral persistence and immune evasion through reseeding latent infection of stem cells [75]. This hypothesis remains to be validated. Additionally, as NRF-1 activates many other genes, the more global effects of US27 on host cell transcription remain to be elucidated. Additionally, whether the modulation of CXCR4 contributes to viral extracellular spread remains to be shown.

The rodent CMVs do not encode a US27 homologue. Despite there being five US28 homologues, RhCMV (AY186194.1) does not have a clear US27 homologue either: a clustal omega search of the RhCMV genome suggests rh220 has the closest homology to US27, which only has 30% sequence identity according to Expasy SIM. ChimpCMV (NC_003521.1), however, encodes chCMVgp157, which has 52.8% sequence identity and is likely a genuine US27 homologue. This raises the question of why US27 evolved in HCMV and CCMV. Potentially, US27 may have arisen during the speciation of great apes from other primates and plays a role specific to these hosts.

## 6. Human Cytomegalovirus US28 and Its Homologues

Compared to the other CMV GPCRs, HCMV US28 has been studied extensively and has a myriad of established and putative roles in HCMV infection. This in itself raises a question: do all CMV GPCRs have multiple roles, which have not been discovered, or is US28 really the “Swiss army knife” as it was once described, or have these myriad roles led to more research interest than in other GPCRs?

GPCRs signal by adopting an open conformation, exposing the intracellular face of the protein and allowing G protein binding and downstream signal transduction. The structure of US28 reveals that the protein sits in the closed conformation but can spontaneously adopt the open conformation, allowing for both ligand-induced and constitutive signalling [84].

During lytic infection, both ligand-induced and constitutive signalling occur, activating multiple different signalling pathways through different Gα proteins in a cell-type- and ligand-dependent manner (Table 1). It has been noted multiple times that US28 signals differently in different cell types [85].

US28 signals during both the lytic and latent phases of HCMV infections. This signalling induces binding of β-arrestin proteins to the C terminus of the US28 and internalisation from the plasma membrane, which stops signalling. Differences in US28 functions can be delineated using protein mutations. The R129A point mutation ablates GPCR signalling through Gα protein binding [18], while Y16F ablates cytokine binding [106]. Truncation of the US28 N terminus also eliminates US28 signalling, as well as decreasing trafficking to the plasma membrane [106], while truncation of the C terminus prevents β-arrestin binding and maintains US28 on the cell surface for longer [107]. Ideally, all functions of US28 should be confirmed in comparison with the relevant US28 mutant, as this controls for the effects of protein overexpression [50]. Importantly, US28 signalling in the cell occurs in the context of whole viral infection. This means that although it is useful to study the US28 protein in isolation, the most relevant information will be corroborated in HCMV infection models controlled with relevant US28 mutant viruses.

US28 signalling during lytic infection tends to support viral replication. This occurs through a range of putative mechanisms including MIEP activation, immune evasion, oncomodulation and cell migration, which have been reviewed extensively before [86]. Most recently, US28 was shown to modulate the proliferation of U251 cells via a Gα_q_/_11_-SK1-S1P-JAK-STAT3 signalling pathway [108].

Recently, US28 was found in secreted exosomes from HCMV-infected fibroblasts. This US28 protein can bind chemokines while in the exosome, thereby acting as a chemokine scavenger, which can have meaningful effects on chemokine-induced signalling in other cells [109].

## 7. US28 and Latent Infection

US28 also plays a major role in HCMV latent infection. US28 appears to be necessary for latent infection [50,70] or reactivation from latency [110]. As the same viral mutants have been used by different groups [111], the most likely explanation for these differences in phenotype could be between different model systems.

**Figure 2:**
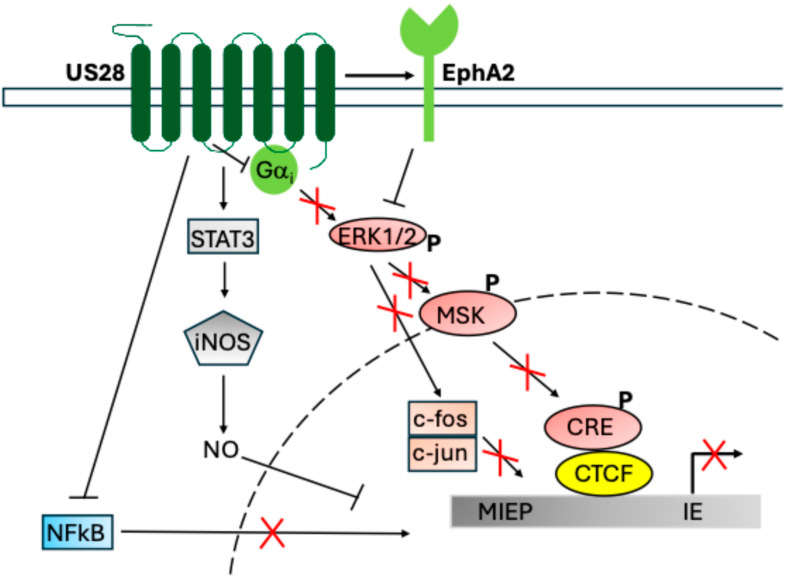
US28 signalling functions during latency, adapted from Elder et al. [118]. US28 signals during latency to repress MIEP activation and downstream HCMV gene expression. Shown here are US28 activation of STAT3-iNOS-NO [112], attenuation of NF-κB [50], and activation of EphA2 [119], which leads to downstream attenuation of ERK-MSK-CREB [50] and fos [120]. NF-κB, p-CREB, and fos-jun (AP-1) directly bind and activate the MIEP in the absence of US28 [50,120]. The mechanism for NO and CTCF [115] suppression of the MIEP is less clear. Red crosses indicate reduced activity in the presence of US28.

Since the initial observation that US28 is required for latent infection, studies have revealed multiple mechanisms that achieve this function. Perhaps central among these is the attenuation of the mitogen-activated protein kinase (MAPK) and NF-κB signalling pathways, reducing phospho-CREB, activator protein-1 (AP-1), Akt, and NF-κB occupancy of the major immediate early promotor (Figure 2). Additionally, US28 signalling via STAT3 activates inducible nitric oxide synthase (iNOS), pushing monocytes towards an immunosuppressive phenotype to maintain latency [112]. US28 signalling has downstream effects on cellular protein expression as well, downregulating the DNA sensors myeloid cell nuclear differentiation antigen (MNDA) and interferon-gamma-induced protein IFI16 (and subsequent interferon-β signalling triggered by these proteins) [113] as well as the repressive transcription factor CTCF [114], which represses MIEP activation through DNA looping [115]. Finally, upregulation of hematopoietic cell lineage-specific protein 1 by US28 increases monocyte cell motility during latency [116] and THP-1 cell adhesion via PLC-β signalling [117].

Most fascinating about this mechanism is the fact that US28 is known to activate the MAPK/NF-κB pathways in lytic HCMV infection, while apparently having the opposite phenotype in latency. This appears to be a cell-type-dependent effect, where US28 signalling differs between monocytes (and monocytic cells) and the same cells after terminal differentiation into macrophages (or macrophage-like cells) and dendritic cells [50,120]. Indeed, earlier evidence already suggested that US28 signals in a cell-type-dependent manner [85]. The mechanisms for US28 attenuation of NF-κB and MAPK are currently unclear; however, two possible routes have been described. Firstly, US28 may interact directly with EphA2, a cell-surface integrin which activates to depress the MAPK pathway [119]; whether EphA2 also attenuates NF-κB is not known. Alternatively, recent structural analysis of the US28 protein suggests that it could bind and act as a Gαi protein “sink”, causing attenuation of signalling by sequestering the Gαi proteins that are needed for signalling [57]. As Gαi signalling could feed into multiple pathways, this may have global effects on cellular signalling such as is mediated by US28 in HCMV latency. Interestingly, this second mechanism notes that US27 ought to have similar functions to US28, although no link between US27 and latent infection currently exists [57]. These two mechanisms are not mutually exclusive and may both occur.

One very novel way to understand the importance of US28 functions in vivo is by deep sequencing US28 from clinical isolates [121]. This approach found key mutations in positions 18–25, N170, and a low rate of variability in positions 314–348 [121]. Molecular dynamics suggest that the N terminal mutations do affect cytokine binding, but importantly, the DRY signalling motif is highly conserved. This agrees with the observation that US28 signalling is required for latency, which is required for viral persistence. Intriguingly, the N170D mutation correlated with higher anti-IE1/2 antibodies and soluble IFN-a in the blood of patients with HIV, which hints at increased reactivation events. N170 is a largely uncharacterised residue in the US28 protein but is found in the third extracellular loop and might interact with binding cytokines.

### US28 Homologues in Primate CMVs

Non-primate CMVs lack a direct US28 homologue, which raises an interesting question: if US28 is required for HCMV latency, how do these CMVs establish latency without US28?

M33, a US28 homologue in murine CMV, appears to play many similar roles to US28 in lytic infection [18], but has not been shown to attenuate signalling pathways in the same way as US28 during latency. However, while HCMV establishes latency in undifferentiated myeloid cells [122], MCMV latency in undifferentiated myeloid cells is controversial [123,124]. MCMV appears to establish latency in other cell types [125,126], so there may be no need for a US28 functional equivalent. M33 deletion in MCMV reduces viral load in the spleen [127], which could be interpreted either as a role in viral latency, or a role in viral tropism in cells that make up the spleen. R33, intriguingly, partially activates Gαi, leading to a constitutive inhibition of CREB [17,42], which is similar in function to US28 during latency [50]. This observation leads to a final possibility: perhaps M33/GP33/R33 attenuate signalling in relevant cell types to maintain latency?

**Figure 3:**
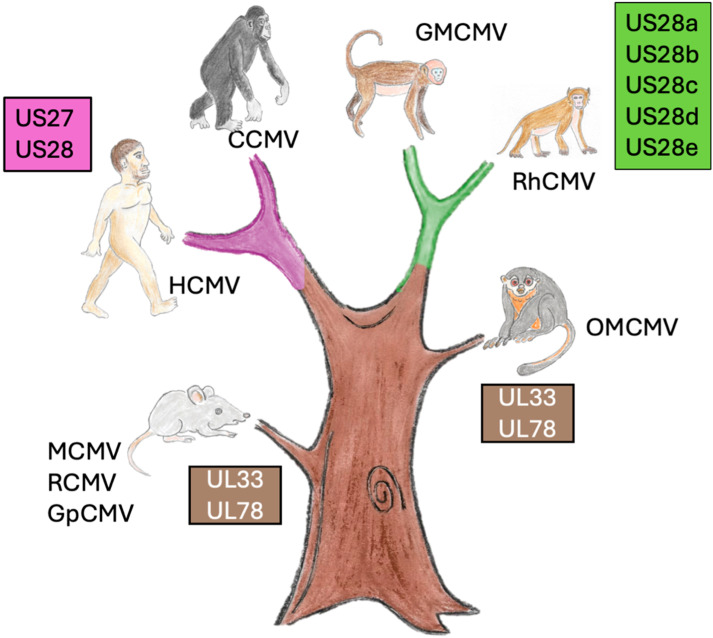
Tree of life showing the evolution of vGPCRs. Every CMV, including those of rodents and New World monkeys, has a homologue of UL33 and UL78 (brown tree trunk). Great apes have US27 and US28 (red branch), while other Old World monkeys have 5 homologues of US28 (green branch).

US28 homologues are found in Old World monkey CMVs, including chimpanzee, golden macaque, and rhesus macaque CMVs, but not in closely related, non-primate CMVs, such as tree shrew CMVs or New World monkey CMVs, such as owl monkey CMVs [128] (Figure 3). This clearly indicates that US28 and its homologues emerged after the Old World/New World monkey split [128]. Chimpanzee CMV (CCMV) has both a US27 and a US28 homologue, like HCMV, while Rhesus CMV [129], golden macaque CMV [129], and cynomolgus macaque CMV [130] all have five. Rh220 can bind fractalkine [131], but little is known about the roles of these US28 homologues beyond this. It would be interesting to see which, if any, of these homologues can functionally replace US28 in an HCMV model latency system. It is likely that these US28 homologues have functions in Old World monkeys that are not required in great apes’ hosts.

## 8. Functions Derived from CMV-Encoded GPCRs in the Virion

Notably, all four HCMV GPCRs have been detected in the virion [35,62,69,120], which likely occurs as GPCRs in the plasma membrane of the viral assembly complex become part of the virion envelope. UL33 and UL78 play a direct role in viral entry [37,58], while US27 and US28 act at the immediate–early stage, before they are expressed [75,87].

If these GPCRs functioned only as virion-delivered proteins at early timepoints, one would expect their expression to be limited to the late stages of infection so that they are incorporated into the virion. This may be true for UL33, which has true late kinetics [35], but also has signalling functions that enhance MIEP activation [17].

US27’s signalling functions may be necessary throughout infection. Its early–late expression would therefore help maintain CXCR4 after virion-delivered protein is depleted. US28 plays key roles during latency, requiring both virion-delivered and expressed protein to establish and maintain latency [120]; however, why it needs to be expressed early during lytic infection is unclear. The kinetics of UL78, which is expressed early in infection [51] but is only known to play a role in viral entry [58], remain a mystery.

## 9. Inhibitors and Drugs Targeting GPCRs as Possible Therapeutic Options

Given their localisation to the cell surface and importance in the HCMV pathogenesis, HCMV-encoded GPCRs have been emerging as attractive targets to treat infections [50,132,133,134,135,136,137,138,139,140]. However, in order to design drugs to target GPCR activities, the mechanisms of action need to be known. The mechanism of action for UL78 is currently unknown, while UL33 appears to facilitate tropism in the absence of its signalling activity. This makes these two proteins currently difficult to target as therapies against HCMV.

US28, on the other hand, is much better characterised and both small and large molecule therapies have been developed against US28 (Table 2). These include both antagonists (which block chemokine-mediated signalling) and inverse agonists (which stop constitutive signalling as well) [50,134,140,141,142,143]. None of the small molecule inhibitors which target US28 have been refined for specific US28 binding, but VUF2274 can inhibit US28 sufficiently to reverse HCMV latency and allow targeting of latently infected cells using a “kick-and-kill” approach [50]. Alternatively, this kick-and-kill approach has also been achieved using US28 targeting inverse agonistic nanobodies [144] or nanobodies which degrade the US28 protein [145]. The US28 protein on the cell surface is already a target for host antibodies during latency [146], and so is an ideal target for nanobodies. As better-refined inverse agonists of US28 are developed, the possibility of kick and kill against US28 is becoming a reality.

Another option is F49A-FTP, a mutant of CX3CL1 with an F49A point mutation, which greatly biases binding to US28 over the host chemokine receptor CX3CR1 [147]. F49A is genetically conjugated to pseudomonas exotoxin, a protein that triggers cell death via translation inhibition [140]. This fusion toxin protein can target HCMV-infected cells in models of lytic [147] and latent [148] infection, as well as in ex vivo cadaverous lungs [149]. Further refinements to F49A-FTP show promising increases in affinity to US28 [150], suggesting possible clinical uses in the future.

**Table 2:** Targets against US28.

**Small Molecules Discovered to Act on US28**				
	**Type**	**Target**	**Mechanism**	**References**
VUF2274	Antagonist and inverse agonist	CCR1 CCL5	Inhibits the PLC-β signalling pathway by sterically blocking chemokine binding and lowering the US28 constitutive signalling activity	[17,136,151,152]
Methiothepin/octoclothepin	Antagonist or partial inverse agonist	CCL5	Blocks CCL5 and inhibits US28 constitutive activity	[16,88,141,153,154]
Hydroisoquinoline-based	Antagonist	CX_3_CR1	Inhibits US28 constitutive activity (p42/p44 mitogen-activated protein kinase (MAPK) and p38 MAPK-dependent pathways)	[153]
Flavonoids	Inverse agonist	---	Inhibit US28 constitutive activity	[155]
Biphenyl amide	Inverse agonist	---	Inhibit US28 constitutive activity	[156]
**Macromolecules Designed to Target US28 (Human Cytomegalovirus)**				
	**Type**		**Mechanism**	**References**
F49A-FTP	Fusion toxin protein (FTP)		A modified CX_3_CL1 which binds US28 with high affinity and targets PE to kill infected cells	[140,148,149]
Bivalent nanobody	Inverse agonist		Displaces CCL5 binding to US28 and prevents constitutive activation of NF-κB and inositol triphosphate (IP3) accumulation. Impairs US28-enhanced tumour growth in vitro and in vivo	[145,157]

PLC: phospholipase C, FTP: fusion toxin protein, PE: pseudomonas exotoxin A.

## 10. Conclusions

Cytomegalovirus-encoded G-protein-coupled receptors play crucial roles in viral pathogenesis, cell spread, cell signalling, and latency. While US28 is well characterised for its involvement in chemokine signalling and latency, the roles of other vGPCRs remain less understood. Indeed, many non-HCMV-encoded vGPCRs have no defined roles. The diversity of vGPCRs among different CMVs presumably reflects their adaptation to host-specific environments. Understanding these differences is necessary for understanding the relevance of animal models in CMV research. Therapeutic strategies targeting US28 are showing promise, and further studies into other GPCRs may elucidate useful future antiviral targets.

## Figures and Tables

**Table 1:** Summary of US28 signalling properties during lytic infection, adapted from [86]. Abbreviations: PLC (phospholipase C), NF-κB (nuclear factor kappa-B), SRF (serum response factor), CREB (cyclic adenosine monophosphate response element binding protein), NFAT (nuclear factor of activated T cells), VEGF (vascular endothelial growth factor), MAPK (mitogen-activated protein kinase), COX2 (cyclooxygenase-2), IL-6 (interleukin 6), JAK (janus kinase), Akt (protein kinase B), PKM2 (pyruvate kinase M2), STAT3 (signal transducer and activator of transcription 3), ERK (extracellular signal-regulated kinase), FAK (focal adhesion kinase), eNOS (endothelial nitric oxide synthase).

Cell System	Ligand	Downstream Signalling and Phenotypes	References
Cos-7 cells	Constitutive but inhibited by CX3CL1 binding	PLC and NF-κB via Gαq	[87,88]
Cos-7 cells	Constitutive but antagonised by CCL5 binding	PLC and NF-κB via Gαo and Gαq/11	[89]
Cos-7 cells	Constitutive	SRF via Gαo and Gαq11 (inhibited by Gα16)	[90]
Infected fibroblasts	Constitutive	PLC via Gαq	[91]
HEK293T	Constitutive	CREB/NFAT a via Gαq	[92]
NIH-3T3 and infected U373	Constitutive	MAPK-induced VEGF-β secretion via Gαq	[93]
NIH-3T3 and infected fibroblasts	Constitutive	NF-κB-induced COX2 and VEGF via Gαq	[94]
NIH-3T3 and HEK293T, infected U373MG	Constitutive	NF-κB induction of IL6, VEGF secretion inducing JAK c/STAT3	[95]
NIH-3T3 and HEK293T; infected fibroblasts and U373MG	Constitutive	β-catenin via both Gα12 and Gαq together	[96]
Infected HASMC, U373MG, HFFs, and HUVECs	Constitutive	PLC-β via Gαq and Gα11 in all cell types tested	[85]
U251 and NIH-3T3	Constitutive	VEGF secretion and HIF1-α activation with Akt and PKM2	[97]
K562	CCL2 and CCL5	Calcium release	[98]
HEK293T and infected HUVECs	CCL7 and CCL5	Calcium release and MAPK via Gα16	[99]
Infected fibroblasts	CCL2 and CCL5	Calcium release	[100]
Infected arterial SMCs	CCL5 (inhibited by CX3CL1)	Chemotaxis via Gα12/13	[101,102,103,104]
Mouse macrophages	CX3CL1 (inhibited by CCL5)	Chemotaxis via Gαq	[103]
Non-proliferating hippocampal cells and HUVECs	CCL5	“Invasive phenotypes” via STAT3, AKT, ERK1/2, FAK, Src, and eNOS	[105]
Infected HASMC and HFFs	CCL5	Calcium release via Gα12/13	[85]

## Data Availability

No new data were generated or analysed in support of this review.
